# Repertoire Development and the Control of Cytotoxic/Effector Function in Human *γ* *δ* T Cells

**DOI:** 10.1155/2010/732893

**Published:** 2010-04-13

**Authors:** Elizabeth M. Urban, Andrei I. Chapoval, C. David Pauza

**Affiliations:** ^1^Institute of Human Virology, University of Maryland, School of Medicine, 725 W. Lombard Street, Baltimore, MD 21201, USA; ^2^Department of Otorhinolaryngology, University of Maryland, School of Medicine, 725 W. Lombard Street, Baltimore, MD 21201, USA

## Abstract

T cells develop into two major populations distinguished by their T cell receptor (TCR) chains. Cells with the *α* *β* TCR generally express CD4 or CD8 lineage markers and mostly fall into helper or cytotoxic/effector subsets. Cells expressing the alternate *γ* *δ* TCR in humans generally do not express lineage markers, do not require MHC for antigen presentation, and recognize nonpeptidic antigens. We are interested in the dominant V*γ*2V*δ*2+ T cell subset in human peripheral blood and the control of effector function in this population. We review the literature on *γ* *δ* T cell generation and repertoire selection, along with recent work on CD56 expression and defining a cytotoxic/effector lineage within the phosphoantigen-reactive V*γ*2V*δ*2 cells. A unique mechanism for MHC-independent repertoire selection is linked to the control of effector function that is vital to the role for *γ* *δ* T cells in tumor surveillance. Better understanding of these mechanisms will improve our ability to exploit this population for tumor immunotherapy.

## 1. Introduction

The idea of using the immune system to combat cancer dates back to 1890, when Paul Ehrlich proposed vaccines against cancer in the wake of various successful immunizations to protect against microbial diseases. The importance of immune surveillance against tumor emergence and progression was reinforced with the observation that immune deficiency states, including iatrogenic immune suppression, severe combined immunodeficiency (SCID), common variable immunodeficiency (CVID), and acquired immunodeficiency syndrome (AIDS), greatly increased patient susceptibility to many types of malignancies [1–4]. Targeting the immune system to combat tumors is in principle, a promising therapeutic strategy [5] although progress has been slow and success is limited. Malignant cells are often difficult to distinguish from normal cells making it difficult to achieve acceptable therapies and there are few schemes for generating immune treatments with sufficient potency to overcome the tumor burden.

The recent discovery of tumor associated antigens, expressed at higher levels or uniquely expressed by tumors cells, provided a means for targeting immune responses to specific malignancies [5, 6]. Efforts have focused on generating major histocompatibility (MHC)-restricted, tumor specific *α* *β* T cells, through vaccination, ex vivo activation or expansion of cytotoxic lymphocytes, or various methods of redirected cytolysis. The efficacy of T cell immunotherapies continues to be limited because tumor neoantigens are usually weak immunogens except in some cases of viral transformation where virus antigens are expressed on malignant cells. Further, MHC tumor-associated antigens or costimulatory molecules can be downregulated to evade detection and tumors can kill or inactivate responding T cells [7–9]. 

Alternatives to conventional T cell responses might include the use of innate-like lymphocytes, such as *γ* *δ* T cells, which have non-MHC restricted recognition of tumor cells. The *γ* *δ* T cell population was first described in 1986 with reports of a new heterodimeric T cell receptor [10] that was associated with CD3 [11]. Rapid growth and evolution of this field lead to the identification of important roles for *γ* *δ* T cells in immune regulation, response to infectious disease, and participation in tumor surveillance. A large and growing interest centers on the possibility of exploiting *γ* *δ* T cells for cancer immunotherapy [12–17]. However, key questions about the activation and cytotoxic activities of these cells remain unanswered, especially regarding the mechanisms controlling cytotoxic effector activities that are limited to a subset or lineage of cells found in adult human beings.

## 2. *γ* *δ* T Cell Development

**Figure 1:**
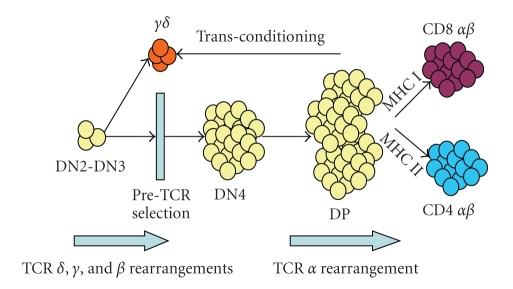
T Cell Development. *γ* *δ* T cells most often arise from the CD4−CD8− (double negative, DN) stages of thymocyte development as a result of successful rearrangements of both the **γ** and **δ** TCR chains. Little, if any, proliferation occurs between these steps, thus limiting diversity. Signals delivered in *trans *from the CD4+CD8+ (double positive, DP) population is crucial for the development of effector functions in *γ* *δ* cells. Figure adapted from Hayday and Pennington [18].

Most of our knowledge about T cell development and especially about the evolution of cytotoxic *γ* *δ* T cells comes from murine studies. T cells develop normally from pluripotent precursor cells in the thymus. A complex series of signaling events direct developing thymocytes to become either *α* *β* or *γ* *δ* T cells (Figure 1). Most progenitors entering the thymus, first from fetal liver and later from bone marrow, are “double negative” (DN) expressing neither CD4 nor CD8, the lineage markers of *α* *β* T cells [19, 20]. Thymocytes progress through at least 4 DN stages [21, 22]. Murine *γ* *δ* T cells emerge mostly from the DN2 and DN3 stages, consistent with their predominantly CD4−CD8− phenotype in the periphery. This is also true for humans, though some plasticity remains even late into *α* *β* differentiation [23]. 

Somatic rearrangement of genes encoding the TCR chains *δ*, *γ*, and *β* begins in DN2 [24]. Several lines of evidence suggest the Tcrd locus (encoding the *δ* TCR chain) rearranges before other TCR genes. In SCID mice, T cell development is arrested just after recombination at Tcrd [25]. In humans, the earliest thymic progenitors (CD34+CD1a−) have a rearranged Tcrd locus, while the Tcrb locus remains in germline configuration [26]. Additionally, partial allelic exclusion is evident at the Tcrg locus [27], indicating that *γ* chains are pairing with preexisting *δ* chains. 

Successful recombination of *δ* and *γ* chains leads to expression of *γ* *δ* TCR on the cell surface. Here, signaling events through the *γ* *δ* TCR are thought to drive the developing thymocytes away from the CD4+CD8+ “double positive” (DP) stage of *α* *β* maturation and onto the *γ* *δ* track. Successful rearrangement of the TCR *β* chain allows pairing of the *β* chain with a surrogate pre-TCR*α* chain, forming the pre-TCR. Signaling through this pre-TCR allows survival, extensive proliferation, and differentiation towards the DP stage of *α* *β* development, followed by rearrangement of the Tcra locus [18]. Great diversity is generated in the *α* *β* population by expansion of pre-TCR expressing cells since multiple daughter cells, bearing identical *β* chains, will then rearrange unique *α* chains and expand repertoire.

There is no evidence for a pre-TCR in *γ* *δ* T cell development [28], indicating that commitment to the *γ* *δ* fate requires a complete TCR with *γ* and *δ* chains successfully rearranged and paired. Little proliferation occurs during *γ* and *δ* chain rearrangement [28]. The requirement for two successful recombination events before *γ* *δ* TCR expression biases thymocytes towards a *α* *β* T cell fate since only the *β* chain needs to be rearranged before expansion of the population, and limits diversity of the *γ* *δ* population [29].

In *α* *β* T cells, negative selection prevents autoimmunity by deleting or inactivating cells that express a self-reactive TCR. Selection is accomplished when self ligands are expressed by medullary thymic epithelial cells [30]. For *γ* *δ* T cells, the criteria for negative selection are poorly defined. Many of the known *γ* *δ* T cell antigens in both humans and mice are self or ubiquitous molecules, making negative selection for these cells more complex perhaps using subtle differences in the strength of signal delivered through the TCR/CD3 complex.

Positive selection in *α* *β* T cells restricts the TCR repertoire by selecting for cells that recognize MHC class I or II molecules. Selection also defines lineage, marked by expression of CD4 or CD8, depending on whether MHC Class I or II molecules are recognized by the **αβ** TCR [31]. Cells recognizing MHC class I downregulate expression of CD4, become single positive for CD8, and evolve as cytolytic effectors. Those cells recognizing MHC class II will downregulate expression of CD8, become single positive for CD4, and adopt cytokine-secreting helper functions [32]. Additional signals, received either during development or upon activation, push *α* *β* T cells down other paths including Th1, Th2, Th17, and regulatory lineages, each with distinct functions. 

For *γ* *δ* T cells, evidence suggests that signaling through the expressed TCR is important for commitment to the *γ* *δ* lineage, but whether this is accomplished through ligand-dependent or independent signaling is unknown [18]. Thymic signals may also affect function. It was discovered recently that a nonclassical MHC molecule T10/T22 binds directly to mouse *γ* *δ* T cell heterodimers mainly in the CDR3 region [33–35]. Mice lacking expression of T10 or T22 develop normal numbers of *γ* *δ* T cells able to recognize these ligands, suggesting that ligand-dependent selection may not be required for differentiation [36]. While the number of *γ* *δ* T cells remained unchanged in T10/T22 knock-outs, the cells are altered functionally. Those that developed in the presence of T10/T22 secreted IFN-*γ* upon activation, while those that developed without antigen secreted IL-17 [36]. Another example of the importance for thymic signaling is seen in the FvB·Tac strain of mice, where the epidermal V*γ*5V*δ*1 TCR repertoire is depleted [37]. The FvB strain defect has been mapped as a mutation in the SkinT gene. SkinT is an Ig superfamily member that encodes a protein in the butyrophilin family. This protein positively selects epidermal *γ* *δ* T cells; a selection does not occur in the mutant FvB strain and the repertoire has a lower frequency of V*γ*5V*δ*1+ cells [38].

In transconditioning [39–41], the functions of *γ* *δ* T cells are partly dependent on the presence of *α* *β* double positive (DP, CD4+CD8+) T cells in the thymus. Mice either lacking the Tcrb gene locus or unable to make the pre-TCR*α* have few double positive *α* *β* cells present in the thymus. *γ* *δ* T cells arising in these mice show altered gene expression profiles [41]. Activated splenic *γ* *δ* T cells in these mice adopt a regulatory phenotype, resembling skin-resident *γ* *δ* cells rather than the usual cytotoxic, IFN-*γ* producing effector phenotype [40]. Since neither *γ* *δ* T cells nor DN2 cells have a cell-autonomous requirement for TCR *β*, these effects are thought to be *in trans * [41].

In 2009, Ribot et al. reported that developing *γ* *δ* T cells in mice could be separated into populations based upon CD27 expression [42]; differences were apparent even at early stages of embryonic development (days 14-15). CD27+ *γ* *δ* T cells that engaged the ligand CD70 became effector cells able to secrete IFN-*γ*. Cells that did not express CD27 developed a regulatory phenotype and secreted IL-17. These data suggested that distinct lineages exist among *γ* *δ* T cells, not unlike CD8 and CD4 *α* *β* T cells, and lineage differences impact effector functions.

Overall, evidence suggests that signals received in the thymus can alter the function of developing *γ* *δ* T cells in mice, either through the expressed TCR or through transconditioning events. Much less is known about the distinct developmental stages and unique signaling requirements for human *γ* *δ* T cells and CD27 expression was noted [43] in cytotoxic and noncytotoxic subpopulations of V*γ*2V*δ*2+ cells, indicating it may not be a strict marker for cytotoxic subsets in man.

## 3. V*γ*2V*δ*2 T Cells

Human *γ* *δ* T cells can be subdivided into two main populations based upon **δ** chain expression. *γ* *δ* T cells expressing the V**δ**1 chain are most often found in mucosal tissues, where they are thought to be involved in maintaining epithelial tissue integrity in the face of damage, infection, or transformation [44, 45]. A second population of *γ* *δ* T cells expresses the V**δ**2 chain and makes up about 1%–10% of circulating lymphocytes in healthy human adults [46]. The V**δ**2 chain pairs almost exclusively with V**γ**2 (called V**γ**9 in an alternative nomenclature). The V**γ**2V**δ**2 pairing is only present in humans and nonhuman primates [44, 47].

*γ* *δ* T cells are often termed innate-like lymphocytes, due to their rapid, antigen-triggered responses, and lack of classical MHC restriction. However, they possess a TCR composed of rearranging germline elements [46], require antigen presentation [48], and undergo peripheral selection [49], arguing they should be classified as components of adaptive immunity. Functionally, they demonstrate cytotoxic responses against cells infected with a variety of viruses, bacteria, or protozoa and they also recognize and kill many human tumors [44, 50]. Cytotoxicity is mediated in much the same manner as for **αβ** T cells, namely, through perforin/granzyme and Fas/FasL pathways or the production of TNF-*α* [51, 52].

*γ* *δ* T cells are preserved evolutionarily in all jawed vertebrates [53] indicating that their role in immune defense is not redundant [44]. Indeed, the **αβ** and *γ* *δ* T cell populations recognize vastly different types of antigens. **αβ** T cells recognize nonself peptide fragments presented by MHC molecules. *γ* *δ* T cells, on the other hand, recognize a wide variety of self-antigens including stress molecules like MICA and MICB, heat shock proteins, and intriguingly, nonpeptidic metabolites of isoprenoid biosynthesis [54–58]. They do not require conventional antigen presentation in the context of MHC [59]. 

The differences in antigen recognition and specificities between the two T cell types define their unique roles in immunity. Studies using both **αβ** and *γ* *δ* T cell-depleted mice showed qualitative and quantitative differences in clearance of infections [60, 61]. Human patients with microbial diseases, such as tuberculosis or malaria, often have large expansions of the peripheral *γ* *δ* T cell subset, sometimes comprising up to 80% of all T cells [62–64]. In the case of malaria, these expanded cells are believed to mediate pathogen elimination. Some viral infections, such as Hepatitis C Virus and Coxsackie B, induce high numbers of *γ* *δ* T cells that home to the site of infection and contribute to pathology [50, 65]. Human Immunodeficiency Virus type 1 (HIV-1) infection results in rapid and specific depletion of the peripheral V**δ**2 T cell population with consequences for the host's ability to resist intercurrent infections [66, 67]. Interestingly, *γ* *δ* T cell levels and activity are high among patients who have natural control of HIV without the use of antiretroviral therapy [68]. These unique protective or pathogenic responses of *γ* *δ* T cells in diverse infectious diseases highlight their unique roles in immunity.

The **γ** and **δ** TCR chains share an Ig-like structure similar to the **αβ** T cell receptor; chain expression is determined by randomly rearranging V, (D) and J segments. In the case of **γ** and **δ** chains, the germline repertoire is restricted severely, due to a limited number of rearranging elements [69]. For instance, the Tcrb locus has 48 functional V and 13 functional J elements, compared to the 8 V and 5 J elements of the Tcrg locus [70]. Much of the CDR3 region diversity is due to N nucleotide addition at the V-J and V-D-J junctions [46]. Due to multiple D segment rearrangements in the **δ** chain, actual diversity may be even greater than that seen in **αβ** T cells [71, 72]. Despite the potential for diversity, greater than 60% of circulating *γ* *δ* T cells bear the V**γ**2V**δ**2 TCR [73, 74], suggesting either restricted chain pairing or a selective chronic expansion of this cell population. Studies comparing the genotypes of *γ* *δ* clones derived from the thymus with those derived from peripheral blood found that thymic clones possessed nearly all possible V**γ**-V**δ** combinations, with the V**γ**2V**δ**2 pairing making up only 5%, indicating that physical restriction on chain pairing was not a critical factor [46]. Nearly all peripheral *γ* *δ* T cells acquire memory markers by the time an individual reaches 2 years of age [75, 76]. These data suggest that selective activation and expansion in response to ubiquitous or self-antigens is the mechanism responsible for overrepresentation of the V**γ**2V**δ**2 T cell subset among adults [71, 77].

**Figure 2:**
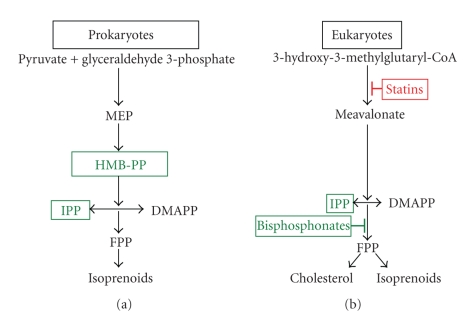
Human V*γ*2V*δ*2 T cells respond to stimulatory phosphoantigens produced during bacterial or mammalian isoprenoid synthesis. Isoprenoid synthesis in many prokaryotes and protists produces the intermediate (*E*)-4-Hydroxy-3-methyl-but-2-enyl pyrophosphate (HMBPP) from pyruvate and glyceraldehyde 3-phosphate via the erythritol 4 phosphate pathway. Eukaryotes use the mevalonate pathway that produces isoprenoids and colesterol. HMBPP and IPP are phosphoantigens; HMBPP is unique to the erythritol 4 phosphate pathway while IPP is produced in both pathways. Phosphoantigens stimulate cytokine secretion and cytotoxicity in human V*γ*2V*δ*2 T cells. Statins block the eukaryotic pathway prior to mevalonate synthesis, and decrease the production of IPP. Bisphosphonates are a class of drugs that inhibit farnesyl pyrophosphate synthase and cause the accumulation of IPP in mammalian cells. When mammalian cells of myeloid origin and some tumor cells are treated with bisphosphonate, they are more stimulatory for V*γ*2V*δ*2 T cells due to increased IPP production.

V**γ**2V**δ**2 T cells respond to low-molecular weight, nonpeptidic, phosphate-containing molecules termed phosphoantigens [78, 79]. The best characterized of these antigens is isopentenyl pyrophosphate (IPP) [54], a substrate in the mevalonate pathway for cholesterol synthesis (Figure 2) in eukaryotes and some bacteria [80]. Physiologic levels of IPP, however, are not stimulatory [81]. A more potent antigen is (*E*)-4-Hydroxy-3-methyl-but-2-enyl pyrophosphate, or HMBPP [82], a substrate in the nonmevalonate pathway found in plants and prokaryotic organisms [83, 84]. The difference in potency is thought to provide a mechanism for self-recognition, wherein *γ* *δ* T cells respond to local bacterial infections, but not to normal tissues [85].

Phosphoantigen stimulation causes a polyclonal expansion of V**γ**2V**δ**2 T cells and selects for the V**γ**2-J**γ**1.2 rearrangement but does not select specific complementary determining region 3 sequences (CDR3). Spectratyping analysis of the V**γ**2 chain demonstrated a strong bias for V**γ**2-J**γ**1.2 rearrangements, reflecting chronic expansion of the population in response to phosphoantigens. Spectratyping of the V**δ**2 chain, on the other hand, showed a normal distribution of chain lengths [86], suggesting less bias in the V**δ**2 chain repertoire and arguing that phosphoantigen recognition is influenced mainly by V*γ* chain sequences.

X-ray crystal structures of a human V**γ**2J**γ**1.2 V**δ**2 T cell receptor revealed a potential positively charged binding pocket made of arginine residues from the V**γ**2 segment and lysine residues from the J**γ**1.2 segment, of which Lys109 appeared to be most important [70]. Site-directed mutagenesis confirmed the importance of these lysine residues, as mutations in the KKIK amino acid stretch encoded in J**γ**1.2 either partially or completely abolished responsiveness to phosphoantigens [87]. No particular rearrangements were selected in the **δ** chain [86], though a conserved hydrophobic residue at position 97 was correlated with antigen recognition [88]. Perhaps the most compelling argument for TCR interactions with phosphoantigen is that transfection of the V**γ**2-J**γ**1.2V**δ**2 TCR into a TCR negative JRT3 T cell line conferred responsiveness to phosphoantigen [89].

Despite evidence implicating the V**γ**2J**γ**1.2 chain in phosphoantigen reactivity, all attempts to show physical interactions between receptor and antigen have been unsuccessful [59]. While neither phosphoantigen-driven activation nor the cytotoxic activity of *γ* *δ* T cells requires MHC molecules, activation does depend upon cell-cell contact with an antigen presenting cell (APC) [48, 90]. Stimulated *γ* *δ* T cells themselves seem to act as APC, but self-activation is not optimal for proliferation responses [91]. Antigen processing by APC is not required; paraformaldehyde-fixed cells are still able to stimulate *γ* *δ* T cell responses [90]. This suggests that an antigen presentation molecule is involved in TCR recognition of phosphoantigen. In fact, tetramers of murine *γ* *δ* TCR bind to cells in an antigen-dependent manner [34] and similar data are emerging for human V*γ*2V*δ*2 TCR [92, 93].

## 4. *γ* *δ* Recognition of Tumor Cells

In addition to their defensive role in microbial infections, *γ* *δ* T cells are cytotoxic against a variety of tumor cell lines including B cell lymphomas, multiple myeloma, and solid tumors of the kidneys, colon, prostate, breast and head and neck [94–97]. Attempts to define a specific antigen among this diverse group of malignancies have pointed to overproduction of metabolic intermediates like IPP, by transformed cells [98]. Repertoire analysis of *γ* *δ* T cells expanded by exposure to IPP or Daudi cells demonstrated very similar V**γ**2 chain repertoire, indicating a substantial overlap of phosphoantigen and tumor cell recognition [99]. Furthermore, bisphosphonates, a class of drugs used to treat certain bone diseases, inhibit farnesyl synthase in the mevalonate pathway, lead to overproduction of IPP [100], and these drugs also stimulate *γ* *δ* T cell proliferation in PBMC cultures. Patients taking these drugs often show significant expansions of the V**γ**2V**δ**2 peripheral T cell subset [14, 101]. 

Not all evidence supports the idea of IPP as a tumor antigen. While all tumors have increased metabolic activity and would therefore produce increased metabolic intermediates such as IPP, not all tumors stimulate V**γ**2V**δ**2 T cells [99]. *γ* *δ* tumor responses are also species specific, despite the fact that tumors from mice or other species will have increased metabolic activity and also produce excess IPP [102]. Moreover, it is not certain that finely tuned tumor surveillance could be accomplished by recognizing an ubiquitous, metabolic intermediate. Freshly isolated *γ* *δ* T cells demonstrate little to no basal cytolytic activity against most tumor cell lines; ex vivo expansion in response to phosphoantigen stimulation is needed to generate broad lytic activity [103, 104]. All of these data imply that the *γ* *δ* TCR is important for recognizing phosphoantigen and proliferating in response to antigen, but its mechanism for discriminating transformed from normal tissues remains unclear.

Given the conflicting evidence for IPP as tumor antigen, an extensive search for conclusive V**γ**2V**δ**2 antigens has been underway. Comparing cell surface markers expressed on tumor cell lines susceptible to *γ* *δ* T cell lysis with those expressed by resistant tumor cell lines suggested that a structure related to the mitochondrial ATP-synthase molecule may be an antigen [105] The F1-ATPase bound directly to the V**γ**2V**δ**2 TCR, as shown by surface plasmon resonance, and binding induced IFN-**γ** and TNF-*α* release from clones. Apolipoprotein A that binds both the TCR and the ATPase [105], enhanced cell activation. While this study provided the first physical evidence for TCR binding to another molecule, the significance of these interactions is unknown. Additionally, blocking of the ATPase by a monoclonal antibody reduced specific tumor cell lysis by *γ* *δ* T cells but did not abolish it, suggesting a role for other cell:cell interactions.

Gene transfer studies demonstrated the dependence of phosphoantigen recognition on the V**γ**2V**δ**2 TCR, as mentioned previously. However, when this system was used to analyze activation by Daudi lymphoma cells, a much weaker response was generated [89]. Studies using Daudi cells fused with melanoma or lymphoma cell lines, resulted in a significant depression of V**γ**2V**δ**2 T cell expansion and a resistance to cytotoxicity, presumably due to expression of MHC class I on the surface of hybrid cells [106]. This suggests a system of self recognition similar to NK cells. There is conflicting evidence regarding antibody blockade of the V**γ**2V**δ**2 TCR. In some reports, TCR blocking resulted in a significant decrease in the ability of cells to lyse tumor targets [107, 108]. Others showed that blocking of the TCR resulted in little or no decrease in cytolysis against a variety of tumor cell types [103, 109], again calling into the question the role for the V**γ**2V**δ**2 TCR and hinting at the presence of other cytotoxicity-mediating molecules on the surface of activated *γ* *δ* T cells.

In addition to the characteristic TCR, *γ* *δ* T cells also possess a variety of NK cell receptors including the activating receptor NKG2D [110], the inhibitory NKG2A, and killer immunoglobulin-like receptors (KIR) [111, 112]; the KIR family of receptors can be activating or inhibitory [113]. NKG2D recognizes the MHC-class I-related molecules MICA and MICB, as well as the UL-16 binding proteins (ULBP), all of which are expressed frequently on transformed cells and deliver activating signals [55, 114]. NKG2D is highly expressed on *γ* *δ* T cells before and after phosphoantigen stimulation. The inhibitory receptors recognize MHC class I molecules on the surface of normal cells and act to inhibit cytotoxic responses, making sure they are directed only against infected or transformed cells. Although *γ* *δ* T cells possess many of the same receptors, NK and *γ* *δ* T cells do not lyse all of the same tumor targets [115] implying unique recognition systems in these two cell types.

It is likely that NK receptors play a role in *γ* *δ* T cell tumor killing [116], but their exact contribution is uncertain and likely influenced by the type of tumor target. As mentioned previously, Daudi cells lack MHC Class I expression on their surface and are highly susceptible to *γ* *δ* T cell lysis [117, 118]. Cytotoxicity is significantly reduced though not abolished, when Daudi cells are engineered to express MHC [106], indicating that inhibitory NK or KIR receptors negatively regulate *γ* *δ* T cell function. In fact, expression of inhibitory KIR on *γ* *δ* T cells correlates with the level of cytotoxicity, a pattern already known for NK and **αβ** T cells [112, 119]. 

Antibody blocking studies directed against the *γ* *δ* TCR indicate that some of the tumoricidal activity seen in expanded cells is TCR-independent [103, 109, 120]. This finding, combined with high levels of NKG2D expressed on expanded *γ* *δ* cells, suggested that activating NKR has a major role in tumor cytolysis [120]. Ligation of NKG2D leads to cytolytic responses, Th1 cytokine secretion, and release of cytotoxic granules [55, 121]. Lytic activity of V**γ**2V**δ**2 cell lines against MICA-expressing targets can be inhibited by up to 50% when blocked by anti-MICA or anti-NKG2D monoclonal antibodies [121]. Unstimulated peripheral blood V**γ**2+ cells also express NKR [112] but still require phosphoantigen stimulation before demonstrating potent lytic activity [121].

While NKR and other costimulatory molecules are likely involved in *γ* *δ* T cell tumor recognition and lysis, cells possessing the specific V**γ**2V**δ**2 TCR are selectively expanded and maintained in peripheral blood and show the highest cytotoxicity toward particular tumor targets. This implies a definitive role for the TCR, but the control over *γ* *δ* T cell-mediated killing remains unclear. The TCR could be the sole signal required for lytic function or might contribute to various activating signals received through other receptors much like NK cells. Alternatively, the TCR could act as a growth factor receptor, much like the TCR in invariant NKT cells [122], recognizing a metabolite and mediating activation of the population without a direct role in tumor cell recognition.

## 5. CD56 as a Marker of Cytotoxicity

As discussed previously, developing thymocytes in the murine system segregated into effector and regulatory *γ* *δ* T cell lineages at early embryonic stages with a prominent role for IL-17 in *γ* *δ* T cells that are naïve to antigen [36, 42]. These distinct subsets were preserved even during antigen stimulation and expansion [42]. In humans, no distinct *γ* *δ* T cell thymocyte subsets are known. When mature cells were expanded with phosphoantigen, potent cytotoxic V**γ**2V**δ**2 effectors could be distinguished from weakly lytic cells by the level of CD56 expression [43]. A low proportion (1%–2%) of adult V*γ* 2V*δ*2 T cells express IL-17 upon phosphoantigen stimulation [123] and might have a regulatory role during responses to infections or tumors. However, little is known about the requirement for *γ* *δ* T cell-produced IL-17 in tumor cytotoxicity.

CD56, also known as (NCAM), is a calcium-independent adhesion molecule, discovered originally in the nervous system [124]. CD56 has been detected on a number of cell types, most notably NK cells and certain cytotoxic T cells (including some *γ* *δ* T cells). CD56 undergoes alternative splicing to generate multiple isoforms depending on cell type and stage of development. Both NK and T cells express exclusively the transmembrane-anchored, 140-kD form [125]. CD56 expression on cytotoxic lymphocytes correlates with lack of MHC restriction, reduced TCR dependence [126], and senescence [127]. The restricted oligoclonal V*β* repertoire present in the CD56+ **αβ** lymphocytes and their memory cell phenotype is consistent with antigen selection and expansion [128]. CD56 does not appear to play a role in the killing activity of CTL, although crosslinking of the molecule does induce cell signaling as evidenced by increased total phosphorylation [129]. In the nervous system, CD56 is involved in cell adhesion through homotypic interactions between CD56-expressing neurons [130]. Studies examining CD56 as an adhesion molecule in NK lysis of tumor cells were inconclusive [129, 131] and the exact function of CD56 in the immune system remains unknown.

Recently, CD56 expression was used as a marker to divide NK cells into highly cytotoxic CD56^dim ^CD16^+^KIR^hi^ and weakly cytotoxic, cytokine-producing CD56^bright^CD16^−^KIR^low^ populations. Once believed to be a uniform effector population, it is now thought that NK cells can be activated and progress along a differentiation path similar to T cells, maturing from CD56^bright^ to CD56^dim ^ upon activation [132].

We and others found that CD56 expression marks a subset of peripheral V**γ**2V**δ**2 T cells that are potently cytotoxic for tumor cells [43, 133]. Following phosphoantigen expansion, about 50% of peripheral *γ* *δ* T cells express CD56 [43]. These cells display potent cytotoxicity against squamous cell carcinoma of the head and neck (SCCHN) cell lines among others, and are resistant to FasL-mediated apoptosis. Expression of CD56 can be induced on CD56 negative fresh *γ* *δ* T cells after treating with IL-2 or IL-15 alone but interestingly, these cells do not gain lytic potential [43] suggesting that stimulation through the TCR and proliferation are together necessary for gain of cytotoxic function.

In V**γ**2V**δ**2 T cells, CD56 is an unusual activation marker. Its expression is not sufficient for cytotoxic activity, since freshly isolated CD56+ *γ* *δ* T cells and cells treated with IL-2 alone are not lytic for tumor cells [43, 104]. After expansion, CD56 expression is a marker for cytotoxicity. When V*γ*2V*δ*2 T cells were expanded by phosphoantigen and separated into CD56+ and CD56− fractions, we found sharp differences in the V*γ*2 chain repertoire among these subsets [134]. Cells in both fractions proliferated rapidly after phosphoantigen/IL-2 stimulation, but the subset expressing CD56 uniquely possessed cytolytic activity against tumor cell targets. Public V*γ*2 chain sequences common to most healthy adults were present in CD56+ or CD56− fractions and their distribution bias was different for each donor, arguing that V*γ*2 chains were not selected to be in the CD56+, cytotoxic subset based on antigen-recognition properties. Further, the reproducible, biased distribution of clones into CD56+ or CD56− fractions showed that control of CD56 expression was not random and the marker did not behave as an activation antigen. The pattern of CD56 expression seems to define a lineage within circulating V*γ*2V*δ*2 T cells. Phosphoantigen triggers proliferation among all cells expressing the V*γ*2J*γ*1.2V*δ*2 TCR, but only the precursor CTL, defined by their capacity to express CD56 after activation, develop cytotoxic effector activities. The mechanisms controlling lineage, whether this occurs before or after TCR expression, and whether mature clones can acquire or lose the capacity to express CD56, all remain as open questions.

The analysis of CD56 expression and TCR sequences on V*γ*2V*δ*2 T cells shows that T cell populations harbor precursors to cytotoxic and noncytotoxic subsets, and the CD56+ subset tends to oligoclonality. The picture for *γ* *δ* T cells is similar to what was observed previously for CD8+ *α* *β* T cells [128, 135] where the CD56+ subset was oligoclonal and required for cytotoxicity. A similar finding was reported previously for CD8+ *α* *β* T cells. In that study [128], human CD8+ *α* *β* T cells were sorted into CD56+ and CD56− fractions before assaying redirected cytotoxicity against murine P815 cells (FcR+) using anti-CD3 monoclonal antibody. The CD56+ fractions had higher value for specific lysis and tended to oligoclonality be compared with CD56− cells from the same donors. Thus, CD56 is a marker for both *α* *β* and *γ* *δ* CTL, and the trend to oligoclonality suggests these cells have been selected and maintained. For V*γ*2V*δ*2 cells, we know that the V*γ*2 chain repertoire is stable for years and possibly decades in healthy donors [99], with a tendency to become more oligoclonal over time. Clearly, selection mechanisms exist to expand and maintain CD56+ subsets and preserve the capacity for CTL function.

The finding that CD56 expression is clonally-restricted but not linked to specific V*γ*2 chains [134] clarifies the mechanism controlling V*γ*2V*δ*2 T cell tumor cytotoxicity but does not solve fundamental problems of tumor cell recognition. Indeed, it is likely that the capacity for expressing CD56 was adopted at an early stage of *γ* *δ* T cell differentiation. Such a mechanism would be consistent with our finding [134] that multiple cells expressing the same V*γ*2 chain nucleotype (indicating that they all derived from the same original clone) segregate together into CD56+ or CD56− subsets. Thus, CD56+ clones within the mature *γ* *δ* T cell repertoire but are not distinguished by TCR specificity. We also know from cell cloning studies [134] that CD56+ cells are heterogeneous with respect to KIR expression. These insights are leading to a view that early lineage marking and heterogeneous expression of KIR limit the proportion of phosphoantigen-responsive V*γ*2V*δ*2+ cells that are capable of tumor cell lysis. In a sense, we are describing NK tumor cell cytolysis except that initial cell activation depends on a rearranged TCR and phosphoantigen recognition. Mature V*γ*2V*δ*2 T cells represent between 1/40 and 1/400 of circulating; CD3+ cells in healthy adults and limitations on the capacity for CTL function may be necessary to prevent lethal autoimmunity during *γ* *δ* responses to infection or malignant transformation. Virtually all V*γ*2V*δ*2 cells produce proinflammatory cytokines IFN-*γ* and TNF-*α* after stimulation, and this function also figures prominently in their immune response activities.

## 6. Summary

Human *γ* *δ* T cells present fascinating challenges to our understanding of T cell selection, repertoire maintenance, and the control of effector functions. Because they have a limited *γ* chain repertoire in adults and they include a high proportion of clines responding to a single antigen, this system permits unique experimental studies not always possible with *α* *β* T cells. An important example is the recent discovery of a cytotoxic effector lineage within the adult population that expresses CD56 and kills tumor cells upon activation. This *γ* *δ* T cell lineage has similarities to CD8+ *α* *β* CTL, but is selected in the absence of lineage marker (CD8) expression and without conventional MHC restriction. In ways that are not yet clear, *γ* *δ* T cells (especially the human V*γ*2V*δ*2+ subset) are selected for response to self-antigens including isopentenylpyrophosphate made by all mammalian cells, without triggering lethal autoimmunity. This produces a T cell subset poised for rapid responses to infected or malignant cells. Much remains to be learned about this interesting subset of T cells, though it is increasingly clear that *γ* *δ* T cells are important for many immune responses and are novel targets for new for new immunotherapies in cancer and infectious disease.

## References

[1] Kersey JH, Spector BD, Good RA (1973). Immunodeficiency and cancer. *Advances in Cancer Research*.

[2] Rabkin CS, Yellin F (1994). Cancer incidence in a population with a high prevalence of infection with human immunodeficiency virus type 1. *Journal of the National Cancer Institute*.

[3] Monforte AD, Abrams D, Pradier C (2008). HIV-induced immunodeficiency and mortality from AIDS-defining and non-AIDS-defining malignancies. *AIDS*.

[4] Salavoura K, Kolialexi A, Tsangaris G, Mavrou A (2008). Development of cancer in patients with primary immunodeficiencies. *Anticancer Research*.

[5] Waldmann TA (2003). Immunotherapy: past, present and future. *Nature Medicine*.

[6] Boon T, Coulie PG, Van den Eynde B (1997). Tumor antigens recognized by T cells. *Immunology Today*.

[7] Dunn GP, Old LJ, Schreiber RD (2004). The immunobiology of cancer immunosurveillance and immunoediting. *Immunity*.

[8] Gattinoni L, Powell DJ, Rosenberg SA, Restifo NP (2006). Adoptive immunotherapy for cancer: building on success. *Nature Reviews Immunology*.

[9] Smyth MJ, Dunn GP, Schreiber RD (2006). Cancer immunosurveillance and immunoediting: the roles of immunity in suppressing tumor development and shaping tumor immunogenicity. *Advances in Immunology*.

[10] Brenner MB, McLean J, Dialynas DP (1986). Identification of a putative second T-cell receptor. *Nature*.

[11] Bank I, DePinho RA, Brenner MB, Cassimeris J, Alt FW, Chess L (1986). A functional T3 molecule associated with a novel heterodimer on the surface of immature human thymocytes. *Nature*.

[12] Dieli F, Gebbia N, Poccia F (2003). Induction of *γ*
*δ* T-lymphocyte effector functions by bisphosphonate zoledronic acid in cancer patients in vivo. *Blood*.

[13] Dieli F, Vermijlen D, Fulfaro F (2007). Targeting human *γ*
*δ* T cells with zoledronate and interleukin-2 for immunotherapy of hormone-refractory prostate cancer. *Cancer Research*.

[14] Kunzmann V, Bauer E, Feurle J, Weissinger F, Tony HP, Wilhelm M (2000). Stimulation of *γ*
*δ* T cells by aminobisphosphonates and induction of antiplasma cell activity in multiple myeloma. *Blood*.

[15] Laggner U, Lopez JS, Perera G (2009). Regression of melanoma metastases following treatment with the n-bisphosphonate zoledronate and localised radiotherapy. *Clinical Immunology*.

[16] Santini D, Martini F, Fratto ME (2009). In vivo effects of zoledronic acid on peripheral *γ*
*δ* T lymphocytes in early breast cancer patients. *Cancer Immunology, Immunotherapy*.

[17] Wilhelm M, Kunzmann V, Eckstein S (2003). *γ*
*δ* T cells for immune therapy of patients with lymphoid malignancies. *Blood*.

[18] Hayday AC, Pennington DJ (2007). Key factors in the organized chaos of early T cell development. *Nature Immunology*.

[19] Scollay R, Wilson A, D’Amico A (1988). Developmental status and reconstitution potential of subpopulations of murine thymocytes. *Immunological Reviews*.

[20] Sanchez MJ, Muench MO, Roncarolo MG, Lanier LL, Phillips JH (1994). Identification of a common T/natural killer cell progenitor in human fetal thymus. *Journal of Experimental Medicine*.

[21] Crispe IN, Moore MW, Husmann LA, Smith L, Bevan MJ, Shimonkevitz RP (1987). Differentiation potential of subsets of CD48 thymocytes. *Nature*.

[22] Godfrey DI, Kennedy J, Suda T, Zlotnik A (1993). A developmental pathway involving four phenotypically and functionally distinct subsets of CD3CD4CD8 triple-negative adult mouse thymocytes defined by CD44 and CD25 expression. *Journal of Immunology*.

[23] Joachims ML, Chain JL, Hooker SW, Knott-Craig CJ, Thompson LF (2006). Human *α*
*β* and *γ*
*δ* thymocyte development: TCR gene rearrangements, intracellular TCR*β* expression, and *γ*
*δ* developmental potential—differences between men and mice. *Journal of Immunology*.

[24] Livak F, Tourigny M, Schatz DG, Petrie HT (1999). Characterization of TCR gene rearrangements during adult murine T cell development. *Journal of Immunology*.

[25] Carroll AM, Bosma MJ (1991). T-lymphocyte development in scid mice is arrested shortly after the initiation of T-cell receptor *δ* gene recombination. *Genes and Development*.

[26] Blom B, Verschuren MCM, Heemskerk MHM (1999). TCR gene rearrangements and expression of the pre-T cell receptor complex during human T-cell differentiation. *Blood*.

[27] Couedel C, Lippert E, Bernardeau K, Bonneville M, Davodeau F (2004). Allelic exclusion at the TCR*δ* locus and commitment to *γ*
*δ* lineage: different modalities apply to distinct human *γ*
*δ* subsets. *Journal of Immunology*.

[28] Passoni L, Hoffman ES, Kim S (1997). Intrathymic *δ* selection events in *γ*
*δ* cell development. *Immunity*.

[29] Taghon T, Yui MA, Pant R, Diamond RA, Rothenberg EV (2006). Developmental and molecular characterization of emerging *β*- and *γ*
*δ*-selected pre-T cells in the adult mouse thymus. *Immunity*.

[30] Takahama Y, Tanaka K, Murata S (2008). Modest cortex and promiscuous medulla for thymic repertoire formation. *Trends in Immunology*.

[31] Chan SH, Waltzinger C, Baron A, Benoist C, Mathis D (1994). Role of coreceptors in positive selection and lineage commitment. *The EMBO Journal*.

[32] He X, Kappes DJ (2006). CD4/CD8 lineage commitment: light at the end of the tunnel?. *Current Opinion in Immunology*.

[33] Crowley MP, Fahrer AM, Baumgarth N (2000). A population of murine *γ*
*δ* T cells that recognize an inducible MHC class Ib molecule. *Science*.

[34] Shin S, El-Diwany R, Schaffert S (2005). Antigen recognition determinants of *γ*
*δ* T cell receptors. *Science*.

[35] Adams EJ, Chien YH, Garcia KC (2005). Structure of a *γ*
*δ* T cell receptor in complex with the nonclassical MHC T22. *Science*.

[36] Jensen KDC, Su X, Shin S (2008). Thymic selection determines *γ*
*δ* T cell effector fate: antigen-naive cells make interleukin-17 and antigen-experienced cells make interferon *γ*. *Immunity*.

[37] Lewis JM, Girardi M, Roberts SJ, Barbee SD, Hayday AC, Tigelaar RE (2006). Selection of the cutaneous intraepithelial *γ*
*δ* T cell repertoire by a thymic stromal determinant. *Nature Immunology*.

[38] Boyden LM, Lewis JM, Barbee SD (2008). Skint1, the prototype of a newly identified immunoglobulin superfamily gene cluster, positively selects epidermal *γ*
*δ* T cells. *Nature Genetics*.

[39] Pennington DJ, Silva-Santos B, Shires J (2003). The inter-relatedness and interdependence of mouse T cell receptor *γ*
*δ* and *α*
*β* cells. *Nature Immunology*.

[40] Pennington DJ, Silva-Santos B, Silberzahn T (2006). Early events in the thymus affect the balance of effector and regulatory T cells. *Nature*.

[41] Silva-Santos B, Pennington DJ, Hayday AC (2005). Lymphotoxin-mediated regulation of *γ*
*δ* cell differentiation by *α*
*β* T cell progenitors. *Science*.

[42] Ribot JC, deBarros A, Pang DJ (2009). CD27 is a thymic determinant of the balance between interferon-*γ*- and interleukin 17-producing *γ*
*δ* T cell subsets. *Nature Immunology*.

[43] Alexander AAZ, Maniar A, Cummings JS (2008). Isopentenyl pyrophosphate-activated CD56 *γ*
*δ* T lymphocytes display potent antitumor activity toward human squamous cell carcinoma. *Clinical Cancer Research*.

[44] Hayday AC (2000). *γ*
*δ* cells: a right time and a right place for a conserved third way of protection. *Annual Review of Immunology*.

[45] Kabelitz D, Glatzel A, Wesch D (2000). Antigen recognition by human *γ*
*δ* T lymphocytes. *International Archives of Allergy and Immunology*.

[46] Casorati G, De Libero G, Lanzavecchia A, Migone N (1989). Molecular analysis of human *γ*/*δ* clones from thymus and peripheral blood. *Journal of Experimental Medicine*.

[47] Rakasz E, MacDougall AV, Zayas MT (2000). *γ*
*δ* T cell receptor repertoire in blood and colonic mucosa of rhesus macaques. *Journal of Medical Primatology*.

[48] Miyagawa F, Tanaka Y, Yamashita S, Minato N (2001). Essential requirement of antigen presentation by monocyte lineage cells for the activation of primary human *γ*
*δ* T cells by aminobisphosphonate antigen. *Journal of Immunology*.

[49] Davodeau F, Peyrat MA, Hallet MM, Houde I, Vie H, Bonneville M (1993). Peripheral selection of antigen receptor junctional features in a major human *γ*
*δ* subset. *European Journal of Immunology*.

[50] Sciammas R, Bluestone JA (1999). TCR*γ*
*δ* cells and viruses. *Microbes and Infection*.

[51] Follows GA, Munk ME, Gatrill AJ, Conradt P, Kaufmann SHE (1992). Gamma interferon and interleukin 2, but not interleukin 4, are detectable in *γ*/*δ* T-cell cultures after activation with bacteria. *Infection and Immunity*.

[52] Barnes PF, Abrams JS, Lu S, Sieling PA, Rea TH, Modlin RL (1993). Patterns of cytokine production by mycobacterium-reactive human T-cell clones. *Infection and Immunity*.

[53] Rast JP, Anderson MK, Strong SJ, Luer C, Litman RT, Litman GW (1997). *α*, *β*, *γ*, and *δ* t cell antigen receptor genes arose early in vertebrate phylogeny. *Immunity*.

[54] Tanaka Y, Morita CT, Tanaka Y, Nieves E, Brenner MB, Bloom BR (1995). Natural and synthetic non-peptide antigens recognized by human *γ*
*δ* T cells. *Nature*.

[55] Bauer S, Groh V, Wu J (1999). Activation of NK cells and T cells by NKG2D, a receptor for stress-inducible MICA. *Science*.

[56] Poccia F, Cipriani B, Vendetti S (1997). CD94/NKG2 inhibitory receptor complex modulates both anti-viral and anti-tumoral responses of polyclonal phosphoantigen-reactive V*γ*9V*δ*2 T lymphocytes. *Journal of Immunology*.

[57] Bukowski JF, Morita CT, Brenner MB (1999). Human *γ*
*δ* T cells recognize alkylamines derived from microbes, edible plants, and tea: implications for innate immunity. *Immunity*.

[58] Fisch P, Malkovsky M, Kovats S (1990). Recognition by human V(*γ*)9/V(*δ*)2 T cells of a GroEL homolog on Daudi Burkitt’s lymphoma cells. *Science*.

[59] Morita CT, Lee HK, Leslie DS, Tanaka Y, Bukowski JF, Märker-Hermann E (1999). Recognition of nonpeptide prenyl pyrophosphate antigens by human *γ*
*δ* T cells. *Microbes and Infection*.

[60] King DP, Ferrick DA, Hyde DM (1999). Cutting edge: protective response to pulmonary injury requires *γ*
*δ* T lymphocytes. *Journal of Immunology*.

[61] Moore TA, Moore BB, Newstead MW, Standiford TJ (2000). *γ*
*δ*-T cells are critical for survival and early proinflammatory cytokine gene expression during murine Klebsiella pneumonia. *Journal of Immunology*.

[62] De Paoli P, Gennari D, Martelli P, Cavarzerani V, Comoretto R, Santini G (1990). *γ*
*δ* T cell receptor-bearing lymphocytes during Epstein-Barr virus infection. *Journal of Infectious Diseases*.

[63] Ho M, Webster HK, Tongtawe P, Pattanapanyasat K, Weidanz WP (1990). Increased *γ*
*δ* T cells in acute Plasmodium falciparum malaria. *Immunology Letters*.

[64] Barnes PF, Grisso CL, Abrams JS, Band H, Rea TH, Modlin RL (1992). *γ*
*δ* T lymphocytes in human tuberculosis. *Journal of Infectious Diseases*.

[65] Tseng CTK, Miskovsky E, Houghton M, Klimpel GR (2001). Characterization of liver T-cell receptor *γ*
*δ* T cells obtained from individuals chronically infected with hepatitis C virus (HCV): evidence for these T cells playing a role in the liver pathology associated with HCV infections. *Hepatology*.

[66] Enders PJ, Yin C, Martini F (2003). HIV-mediated *γ*
*δ* T cell depletion is specific for V*γ*2+ cells expressing the J*γ*1.2 segment. *AIDS Research and Human Retroviruses*.

[67] Hinz T, Wesch D, Friese K, Reckziegel A, Arden B, Kabelitz D (1994). T cell receptor *γ*
*δ* repertoire in HIV-1-infected individuals. *European Journal of Immunology*.

[68] Riedel DJ, Sajadi MM, Armstrong CL (2009). Natural viral suppressors of HIV-1 have a unique capacity to maintain *γ*
*δ* T cells. *AIDS*.

[69] Chien YH, Bonneville M (2006). Gamma delta T cell receptors. *Cellular and Molecular Life Sciences*.

[70] Allison TJ, Winter CC, Fournie JJ, Bonneville M, Garboczi DN (2001). Structure of a human *γ*
*δ*T-cell antigen receptor. *Nature*.

[71] Carding SR, Egan PJ (2002). *γ*
*δ* T cells: functional plasticity and heterogeneity. *Nature Reviews Immunology*.

[72] Chien YH, Konigshofer Y (2007). Antigen recognition by *γ*
*δ* T cells. *Immunological Reviews*.

[73] De Libero G, Casorati G, Giachino C (1991). Selection by two powerful antigens may account for the presence of the major population of human peripheral *γ*/*δ* T cells. *Journal of Experimental Medicine*.

[74] Panchamoorthy G, McLean J, Modlin RL (1991). A predominance of the T cell receptor V*γ*2/V*δ*2 subset in human mycobacteria-responsive T cells suggests germline gene encoded recognition. *Journal of Immunology*.

[75] Davodeau F, Peyrat MA, Hallet MM (1993). Close correlation between Daudi and mycobacterial antigen recognition by human *γ*
*δ* T cells and expression of V9JPC1*γ*/V2DJC*δ*-encoded T cell receptors. *Journal of Immunology*.

[76] De Rosa SC, Andrus JP, Perfetto SP (2004). Ontogeny of *γ*
*δ* T cells in humans. *Journal of Immunology*.

[77] Parker CM, Groh V, Band H (1990). Evidence for extrathymic changes in the T cell receptor *γ*/*δ* repertoire. *Journal of Experimental Medicine*.

[78] Pfeffer K, Schoel B, Gulle H, Kaufmann SHE, Wagner H (1990). Primary responses of human T cells to mycobacteria: a frequent set of *γ*/*δ* T cells are stimulated by protease-resistant ligands. *European Journal of Immunology*.

[79] Tanaka Y, Sano S, Nieves E (1994). Nonpeptide ligands for human *γ*
*δ* T cells. *Proceedings of the National Academy of Sciences of the United States of America*.

[80] Banthorpe DV, Charlwood BV, Francis MJO (1972). The biosynthesis of monoterpenes. *Chemical Reviews*.

[81] Jomaa H, Feurle J, Luhs K (1999). V*γ*9/V*δ*2 T cell activation induced by bacterial low molecular mass compounds depends on the 1-deoxy-D-xylulose 5-phosphate pathway of isoprenoid biosynthesis. *FEMS Immunology and Medical Microbiology*.

[82] Hintz M, Jomaa H, Reichenberg A (2001). Identification of (E)-4-hydroxy-3-methyl-but-2-enyl pyrophosphate as a major activator for human *γ*
*δ* T cells in Escherichia coli. *FEBS Letters*.

[83] Arigoni D, Sagner S, Latzel C, Eisenreich W, Bacher A, Zenk MH (1997). Terpenoid biosynthesis from 1-deoxy-D-xylulose in higher plants by intramolecular skeletal rearrangement. *Proceedings of the National Academy of Sciences of the United States of America*.

[84] Rohmer M, Knani M, Simonin P, Sutter B, Sahm H (1993). Isoprenoid biosynthesis in bacteria: a novel pathway for the early steps leading to isopentenyl diphosphate. *Biochemical Journal*.

[85] Eberl M, Hintz M, Reichenberg A, Kollas AK, Wiesner J, Jomaa H (2003). Microbial isoprenoid biosynthesis and human *γ*
*δ* T cell activation. *FEBS Letters*.

[86] Evans PS, Enders PJ, Yin C, Ruckwardt TJ, Malkovsky M, Pauza CD (2001). In vitro stimulation with a non-peptidic alkylphosphate expands cells expressing V*γ*2-J*γ*1.2/V*δ*2 T-cell receptors. *Immunology*.

[87] Miyagawa F, Tanaka Y, Yamashita S (2001). Essential contribution of germline-encoded lysine residues in J*γ*1.2 segment to the recognition of nonpeptide antigens by human *γ*
*δ* T cells. *Journal of Immunology*.

[88] Yamashita S, Tanaka Y, Harazaki M, Mikami B, Minato N (2003). Recognition mechanism of non-peptide antigens by human *γ*
*δ* T cells. *International Immunology*.

[89] Bukowski JF, Morita CT, Tanaka Y, Bloom BR, Brenner MB, Band H (1995). V*γ*2V*δ*2 TCR-dependent recognition of non-peptide antigens and Daudi cells analyzed by TCR gene transfer. *Journal of Immunology*.

[90] Morita CT, Beckman EM, Bukowski JF (1995). Direct presentation of nonpeptide prenyl pyrophosphate antigens to human *γ*
*δ* T cells. *Immunity*.

[91] Brandes M, Willimann K, Moser B (2005). Professional antigen-presentation function by human *γ*
*δ* T cells. *Science*.

[92] Sarikonda G, Wang H, Puan KJ (2008). Photoaffinity antigens for human *γ*
*δ* T cells. *Journal of Immunology*.

[93] Wei H, Huang D, Lai X (2008). Definition of APC presentation of phosphoantigen (E)-4-hydroxy-3-methyl-but-2-enyl pyrophosphate to Vgamma2Vdelta 2 TCR. *Journal of Immunology*.

[94] Corvaisier M, Moreau-Aubry A, Diez E (2005). V*γ*9V*δ*2 T cell response to colon carcinoma cells. *Journal of Immunology*.

[95] Guo BL, Liu Z, Aldrich WA, Lopez RD (2005). Innate anti-breast cancer immunity of apoptosis-resistant human *γ*
*δ*-T cells. *Breast Cancer Research and Treatment*.

[96] Kunzmann V, Wilhelm M (2005). Anti-lymphoma effect of *γ*
*δ* T cells. *Leukemia and Lymphoma*.

[97] Mattarollo SR, Kenna T, Nieda M, Nicol AJ (2007). Chemotherapy and zoledronate sensitize solid tumour cells to V*γ*9V*δ*2 T cell cytotoxicity. *Cancer Immunology, Immunotherapy*.

[98] Gober HJ, Kistowska M, Angman L, Jeno P, Mori L, De Libero G (2003). Human T cell receptor *γ*
*δ* cells recognize endogenous mevalonate metabolites in tumor cells. *Journal of Experimental Medicine*.

[99] Hebbeler AM, Cairo C, Cummings JS, Pauza CD (2007). Individual V*γ*2-J*γ*1.2+ T cells respond to both isopentenyl pyrophosphate and Daudi cell stimulation: generating tumor effectors with low molecular weight phosphoantigens. *Cancer Immunology, Immunotherapy*.

[100] Bonneville M, Fournie JJ (2005). Sensing cell stress and transformation through V*γ*9V*δ*2 T cell-mediated recognition of the isoprenoid pathway metabolites. *Microbes and Infection*.

[101] Kunzmann V, Bauer E, Wilhelm M (1999). *γ*/*δ* T-cell stimulation by pamidronate. *The New England Journal of Medicine*.

[102] Kato Y, Tanaka Y, Tanaka H, Yamashita S, Minato N (2003). Requirement of species-specific interactions for the activation of human *γ*
*δ* T cells by pamidronate. *Journal of Immunology*.

[103] Kabelitz D, Wesch D, Pitters E, Zoller M (2004). Characterization of tumor reactivity of human V*γ*9V*δ*2 *γ*
*δ* T cells in vitro and in SCID mice in vivo. *Journal of Immunology*.

[104] Kato Y, Tanaka Y, Miyagawa F, Yamashita S, Minato N (2001). Targeting of tumor cells for human *γ*
*δ*
T cells by nonpeptide antigens. *Journal of Immunology*.

[105] Scotet E, Martinez LO, Grant E (2005). Tumor recognition following V*γ*9V*δ*2 T cell receptor interactions with a surface F1-ATPase-related structure and apolipoprotein A-I. *Immunity*.

[106] Fisch P, Meuer E, Pende D (1997). Control of a cell lymphoma recognition via natural killer inhibitory receptors implies a role for human V*γ*9/V*δ*2 T cells in tumor immunity. *European Journal of Immunology*.

[107] Bouet-Toussaint F, Cabillic F, Toutirais O (2008). V*γ*9V*δ*2 T cell-mediated recognition of human solid tumors. Potential for immunotherapy of hepatocellular and colorectal carcinomas. *Cancer Immunology, Immunotherapy*.

[108] Viey E, Fromont G, Escudier B (2005). Phosphostim-activated *γ*
*δ* T cells kill autologous metastatic renal cell carcinoma. *Journal of Immunology*.

[109] Ensslin AS, Formby B (1991). Comparison of cytolytic and proliferative activities of human *γ*
*δ* and *α*
*β* T cells from peripheral blood against various human tumor cell lines. *Journal of the National Cancer Institute*.

[110] Rincon-Orozco B, Kunzmann V, Wrobel P, Kabelitz D, Steinle A, Herrmann T (2005). Activation of V*γ*9V*δ*2 T cells by NKG2D. *Journal of Immunology*.

[111] Lafarge X, Pitard V, Ravet S (2005). Expression of MHC class I receptors confers functional intraclonal heterogeneity to a reactive expansion of *γ*
*δ* T cells. *European Journal of Immunology*.

[112] Halary F, Peyrat MA, Champagne E (1997). Control of self-reactive cytotoxic T lymphocytes expressing *γ*
*δ* T cell receptors by natural killer inhibitory receptors. *European Journal of Immunology*.

[113] Vilches C, Parham P (2002). KIR: diverse, rapidly evolving receptors of innate and adaptive immunity. *Annual Review of Immunology*.

[114] Sutherland CL, Chalupny NJ, Cosman D (2001). The UL16-binding proteins, a novel family of MHC class I-related ligands for NKG2D, activate natural killer cell functions. *Immunological Reviews*.

[115] Fisch P, Malkovsky M, Braakman E (1990). *γ*/*δ* T cell clones and natural killer cell clones mediate distinct patterns of non-major histocompatibility complex-restricted cytolysis. *Journal of Experimental Medicine*.

[116] Trichet V, Benezech C, Dousset C, Gesnel MC, Bonneville M, Breathnach R (2006). Complex interplay of activating and inhibitory signals received by V*γ*9V*δ*2 T cells revealed by target cell *β*-microglobulin knockdown. *Journal of Immunology*.

[117] Sturm E, Braakman E, Fisch P, Sondel PM, Bolhuis RLH (1991). Daudi cell specificity correlates with the use of a V*γ*9-V*δ*2 encoded TCR*γ*
*δ*. *Current Topics in Microbiology and Immunology*.

[118] Sturm E, Braakman E, Fisch P, Vreugdenhil RJ, Sondel P, Bolhuis RLH (1990). Human V*γ*9-V*δ*2 T cell receptor-*γ*
*δ* lymphocytes show specificity to Daudi Burkitt’s lymphoma cells. *Journal of Immunology*.

[119] Fisch P, Moris A, Rammensee HG, Handgretinger R (2000). Inhibitory MHC class I receptors on *γ*
*δ* T cells in tumour immunity and autoimmunity. *Immunology Today*.

[120] Wrobel P, Shojaei H, Schittek B (2007). Lysis of a broad range of epithelial tumour cells by human *γ*
*δ* T cells: involvement of NKG2D ligands and T-cell receptor-versus NKG2D-dependent recognition. *Scandinavian Journal of Immunology*.

[121] Das H, Groh V, Kuijl C (2001). MICA engagement by human V*γ*2V*δ*2 T cells enhances their antigen-dependent effector function. *Immunity*.

[122] Godfrey DI, Berzins SP (2007). Control points in NKT-cell development. *Nature Reviews Immunology*.

[123] Li H, Pauza CD (2009). Effects of 15-deoxy-Δ^12,14^-prostaglandin J2 (15d-PGJ2) and rosiglitazone on human V*δ*2^+^ T cells. *PLoS One*.

[124] Cunningham BA, Hemperly JJ, Murray BA, Prediger EA, Brackenbury R, Edelman GM (1987). Neural cell adhesion molecule: structure, immunoglobulin-like domains, cell surface modulation, and alternative RNA splicing. *Science*.

[125] Lanier LL, Testi R, Bindl J, Phillips JH (1989). Identity of Leu-19 (CD56) leukocyte differentiation antigen and neural cell adhesion molecule. *Journal of Experimental Medicine*.

[126] Schmidt RE, Murray C, Daley JF, Schlossman SF, Ritz J (1986). A subset of natural killer cells in peripheral blood displays a mature T cell phenotype. *Journal of Experimental Medicine*.

[127] Lemster BH, Michel JJ, Montag DT (2008). Induction of CD56 and TCR-independent activation of T cells with aging. *Journal of Immunology*.

[128] Pittet MJ, Speiser DE, Valmori D, Cerottini JC, Romero P (2000). Cutting edge: cytolytic effector function in human circulating CD8 T cells closely correlates with CD56 surface expression. *Journal of Immunology*.

[129] Lanier LL, Chang C, Azuma M, Ruitenberg JJ, Hemperly JJ, Phillips JH (1991). Molecular and functional analysis of human natural killer cell-associated neural cell adhesion molecule (N-CAM/CD56). *Journal of Immunology*.

[130] Chuong CM, McClain DA, Streit P, Edelman GM (1982). Neural cell adhesion molecules in rodent brains isolated by monoclonal antibodies with cross-species reactivity. *Proceedings of the National Academy of Sciences of the United States of America*.

[131] Nitta T, Yagita H, Sato K, Okumura K (1989). Involvement of CD56 (NKH-1/Leu-19 antigen) as an adhesion molecule in natural killer-target cell interaction. *Journal of Experimental Medicine*.

[132] Romagnani C, Juelke K, Falco M (2007). CD56^bright^CD16^−^ killer Ig-like Receptor^−^ NK cells display longer telomeres and acquire features of CD56^dim^ NK cells upon activation. *Journal of Immunology*.

[133] Li H, Luo K, Maniar A, Chapoval AI, Salvato MS, Pauza CD CD56 expression and function in Vgamma2 Vdelta2 T cells.

[134] Urban EM, Li H, Armstrong C, Focaccetti C, Cairo C, Pauza CD (2009). Control of CD56 expression and tumor cell cytotoxicity in human V*γ*2V*δ*2 T cells. *BMC Immunology*.

[135] Tokuyama H, Hagi T, Mattarollo SR (2008). V*γ*9V*δ*2 T cell cytotoxicity against tumor cells is enhanced by monoclonal antibody drugs—rituximab and trastuzumab. *International Journal of Cancer*.

